# Cadherin2 (N-cadherin) plays an essential role in zebrafish cardiovascular development

**DOI:** 10.1186/1471-213X-6-23

**Published:** 2006-05-23

**Authors:** Brian Bagatto, Jessie Francl, Bei Liu, Qin Liu

**Affiliations:** 1 Department of Biology, University of Akron, Akron, Ohio, USA

## Abstract

**Background:**

Cadherins are cell surface adhesion molecules that play important roles in development of vertebrate tissues and organs. We studied cadherin2 expression in developing zebrafish heart using *in situ *hybridization and immunocytochemical methods, and we found that cadherin2 was strongly expressed by the myocardium of the embryonic zebrafish. To gain insight into cadherin2 role in the formation and function of the heart, we analyzed cardiac differentiation and performance in a *cadherin2 *mutant, *glass onion *(*glo*).

**Results:**

We found that the *cadherin2 *mutant had enlarged pericardial cavity, disorganized atrium and ventricle, and reduced expression of a ventricular specific marker *vmhc*. Individual myocardiocytes in the *glo *mutant embryos became round shaped and loosely aggregated. *In vivo *measurements of cardiac performance revealed that the mutant heart had significantly reduced heart rate, stroke volume and cardiac output compared to control embryos. Formation of the embryonic vascular system in the *glo *mutants was also affected.

**Conclusion:**

Our results suggest that cadherin2 plays an essential role in zebrafish cardiovascular development. Although the exact mechanisms remain unknown as to the formation of the enlarged pericardium and reduced peripheral blood flow, it is clear that  myocardiocyte differentiation and physiological cardiovascular  performance is impaired when cadherin2 function is disrupted.

## Background

Zebrafish (*Danio rerio*) has emerged as an important model system in the study of vertebrate development due to its external development, transparency of embryos, and its demonstrated utility as a genetic model. The process of heart formation occurs in a similar way in all vertebrates [1]. Heart development in the zebrafish is also rapid, which allows for numerous short-term studies. The primitive heart tube is formed by 22 hours post-fertilization (hpf). The heart is beating and the circulation becomes evident at 24 hpf. By 30 hpf, the heart tube starts to loop to the right side of the embryo. By 36 hpf, chamber boundaries are evident, although molecular markers can distinguish them in the primitive heart tube [2,3]. Looping places both the atrium and the ventricle toward the left of the embryo, however the atrium is further to the left than the ventricle. By 60 hpf, the valves are present and by the fifth day, the heart has assumed its adult configuration, with the atrium sitting dorsally with respect to the ventricle [1,4].

The fundamental plan of the vascular system as it develops in the zebrafish is similar to that of other vertebrates. The overall form of the zebrafish vasculature is established early, before the initiation of circulation, and the pattern of major vessel tracts is reproducible from embryo to embryo [5]. Circulation begins at approximately 24–26 hpf and initially flows through a simple single circulatory loop. Cells from the dorsal margin of the lateral plate migrate medially to form the intermediate cell mass [6]. This mass gives rise to both the endothelia and the major trunk vessels and the first cohort of blood cells [7]. However, many early vasculogenic vessels first appear as a network or plexus of smaller vessels, with little apparent pattern or differentiated identity [8,9]. By 2.5–3 days postfertilization (dpf), the trunk and tail intersegmental vessels are fully formed and by 6 dpf, the overall basic pattern of the vaculature is in place [10].

Although vertebrate cardiovascular development has been well described morphologically, the molecular and physiological mechanisms underlying these events are only beginning to be understood. Results from gene expression pattern and/or functional studies suggest that a variety of molecules, including transcription factors (e.g. Gata4, Nkx2.5), morphogenetic regulators (e.g. Hand2, Pitx2, Xin), endothelial growth factors (e.g. VEGF-A), cardiac specific proteins (e.g. cmlc1, cmlc2, and vmhc), cell adhesion molecule (e.g. cadherin2 and cadherin5, see below), are involved in the cardiac patterning and morphogenesis of the vertebrate heart [3,11-20].

The cadherins are a family of Ca^++^-dependent transmembrane molecules that mediate cell adhesion mainly through homophilic interactions [21-23]. Cadherin2, the first cadherin discovered in the vertebrate nervous system [24], has been shown to be of critical importance in the early differentiation of the vertebrate central and peripheral nervous structures [18,25-30]. Unlike the wide expression of cadherin2, expression of cadherin5 (also called VE-cadherin), is confined to the endothelial cells of both developing and adult vasculature [20,31,32]. Despite the importance of cardiovascular tissue itself and cadherin molecules in animal development, there are only a few published reports on cadherins function in vertebrate cardiovascular development. Zebrafish with mutations in *cadherin2 *(*parachute*, or *pac *mutant, [28]; *glass onion*, or *glo *mutant, [29]) have recently been identified, and their phenotypes studied. However, most of the analysis was concentrated on the central nervous system. So far, detailed information on the developmental profile of cadherin2 expression in the vertebrate cardiac tissue has been performed on only the chick and mice. Moreover, functional analysis was limited almost exclusively to descriptions of anatomical defects in the heart of embryos whose cadherin2 function was blocked [18,33]. We propose to study cardiac differentiation, cardiac performance and formation of the vascular system in the *glo *mutant in order to elucidate the role(s) of cadherin2 plays in the formation and function of the cardiovascular system.

## Results

### Cadherin2 expression in developing zebrafish heart

**Figure 1:**
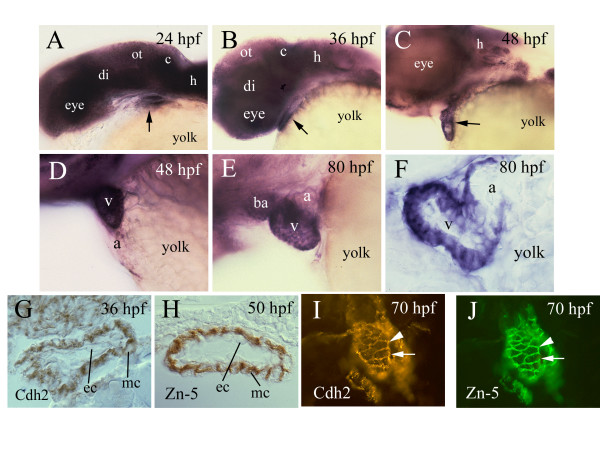
**Cadherin2 expression in developing zebrafish heart**. Anterior is to the left and dorsal is up for panels A-H. Panels A-C are lateral views of the head region of whole mount zebrafish embryos labeled by in situ hybridization with *cadherin-2 *cRNA. The arrow points to the heart. Panels D and E are lateral views of higher magnifications of the heart. Panel F is a parasagittal section of a heart processed for whole mount in situ hybridization. Panels G and H are parasagittal sections of the ventricle processed for cadherin-2 (Cdh2) and Zn-5 immunocytochemical staining, respectively, both showing that the staining is confined mainly to cell membranes of myocardiocytes. Panels I and J show the same cross section of the ventricle (dorsal up) double-labeled with cadherin-2 antibody (panel I) and Zn-5 antibody (panel J). The arrows and arrowheads point to the same cells respectively. Abbreviations: a, atrium; ba, bulbus arteriosus; c, cerebellum; di, diencephalon; ec, endothelium; h, hindbrain; mc, myocardium; ot, optic tectum; v, ventricle.

Cadherin2 expression in the developing zebrafish heart was studied using whole mount *in situ* hybridization and immunocytochemical methods on tissue sections. Strong *cadherin2 *message was detected in the developing zebrafish heart of all stages examined (24 hpf to 80 hpf; Fig. 1). Expression levels were higher in the bulbus arteriosus and the ventricle than in the atrium in whole mount hearts (Fig. 1D and 1E). Examination of sections from the whole mount hearts and tissue sections processed for cadherin2 immunostaining showed that the labeling was confined mainly to the myocardial layer at the stages examined (Fig. 1G). This was confirmed by double labeling experiments using the cadherin2 antibody and an anti-mouse monoclonal antibody Zn-5, which has been shown to recognize differentiating zebfrafish ventricular myocardium (Deborah Yelon, New York University, personal communication; Fig. 1H–J). The difference in cadherin2 staining between the atrium and ventricle was mainly due to the thickness of these structures with the ventricle having a myocardium consisting 2–3 cell layers while the atrium myocardium having a single cell layer (Fig. 1F; [4]).

### Analysis of cardiac performance in *glo *mutant embryos

**Figure 2:**
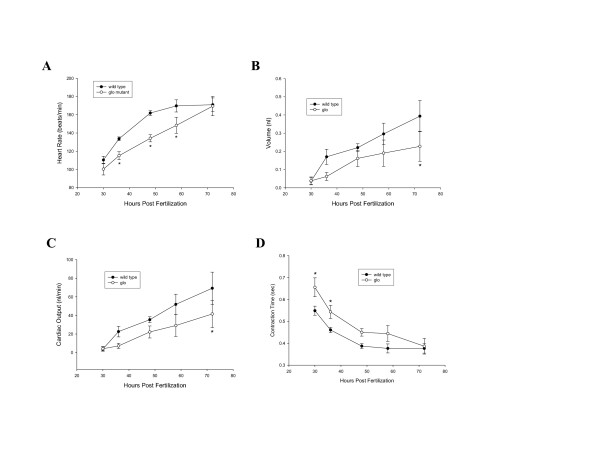
**Measurements of cardiac performance in control and *glo *mutant embryos**. Panel A shows mean heart rate in both groups during development and panel B shows stroke volume in the same larvae. Panel C is the result of the product of the data in panels A and B. Panel D shows the time from end diastole to end systole. Asterisks represent a significant difference between the control and the *glo *embryos during that time of development. All data shown are means ± S.E.M.

The expression pattern of cadherin2 in the embryonic zebrafish heart suggests that this adhesion molecule is involved in the development of normal zebrafish cardiac function. We tested this idea by examining several key cardiac performance parameters: heart rate, stroke volume, cardiac output, and contraction time in control and *glo *mutant 30–72 hpf embryos (Fig. 2). By 30 hpf, these parameters can be readily recorded under an inverted dissection microscope. Mean heart rates of *glo *mutant embryos were lower than controls at this stage, but the difference was not statistically significant (Fig. 2A). The heart rates increased by almost 60% in both groups over the 42-hour measurement period. However, the trajectory of this increase was significantly different between the control and *glo *mutant embryos. Between 30–48 hpf, differences between the mean heart rates of these two groups grew more prominent, with the values significantly lower in the *glo *mutant embryos than the controls at 36 and 48 hpf (Fig. 2A). Interestingly, differences in the heart rates between the two groups became smaller between 48–72 hpf, due to a faster increase in the *glo *mutant heart rates. Although the mean heart rates were still significantly lower for *glo *mutant embryos at 58 hpf, their values were similar to the controls at 72 hpf. Moreover, variations in hearts rate were also significantly higher in the *glo *mutant embryos.

Mean stroke volume in control and *glo *mutant hearts was similar at 30 hpf, and it became larger in both groups as development proceeded (Fig. 2B). In control embryos, stroke volume increased more rapidly during development with the difference showing statistical significance at 72 hpf. This late significance was due, at least partially, to large variations of the stroke volume in both groups. Multiplying stroke volume by heart rate produces cardiac output (Fig. 2C). Mean cardiac output increased in both groups during the 42-hour measurement period with the slope of the increase significantly higher in the control group. As with stroke volume, cardiac output was similar between the two groups at 30 hpf, and the control embryos had higher cardiac output than the *glo *mutant embryos at the remaining recording time points, although their values were not significantly different until 72 hpf. Again, cardiac output variability was high in both groups.

For the majority of the developmental window investigated, the contraction time (time from diastole to systole) for *glo *mutant hearts was slower than controls (Fig. 2D). This difference was statistically significant during the first two measurement times (30 and 36 hpf), but by 72 hpf, the contraction time became similar between the *glo *and control hearts. Moreover, the variation in contraction times was also significantly higher in the *glo *mutant hearts, reflecting the irregular nature of cardiac contractions in this group.

### Gross morphological defects in the heart of the *glo *mutant and *cadherin2* morphants

**Figure 3:**
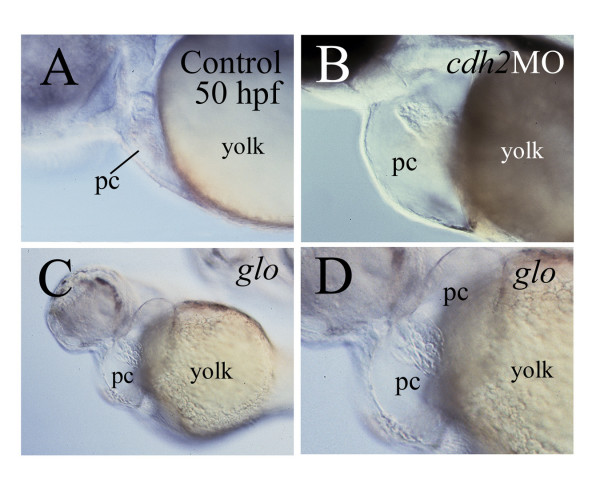
**Enlarged pericardial cavity in *glo *mutant**. Compared to a control embryo (panel A), the pericardial cavity (pc) is much enlarged in a *cadherin2 *morphant (panel B) and a *glo *mutant embryo (panels C and D). All panels are images from live embryos showing lateral views (anterior to the left and dorsal up) of the pericardial cavity and heart. Panel D is a higher magnification of the pericardial cavity showing in panel C.

**Figure 4:**
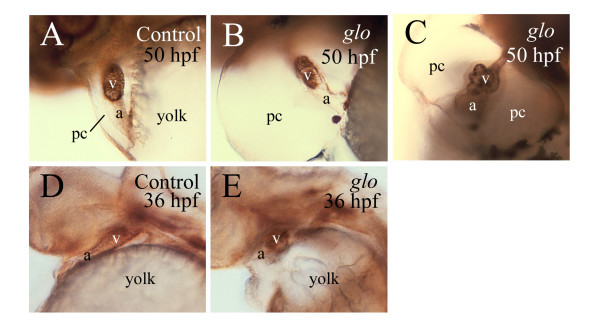
**Gross cardiac morphological defects in *glo *mutant embryos revealed by Zn-5 immunostaining**. All panels show lateral views of the heart of whole mount embryos labeled with the Zn-5 antibody. Anterior is to the left and dorsal is up. Abbreviations are the same as in Figure 1.

The decreased cardiac function in the *glo *mutant embryos was likely caused by morphological changes in the mutant heart. The pericardial cavity was greatly enlarged in all *glo *mutants (Fig. 3C and 3D), in most (264/305, 86.6%) of *cadherin2 *morphants (Fig. 3B), but in only a small number (3/78, 3.4%) of embryos injected with a standard control morpholino oligonucleotide. *glo *mutant embryos become readily distinguishable from their heterozygous and wild type cohorts at 24 hpf by having tail blisters or clubbed tails [29]. At this stage the pericardial cavity of the *glo *mutant embryos was similar in size to control embryos. The *glo *mutant pericardial cavity remained similar in size to control embryos at 28 and 36 hpf (data not shown), but by 50 hpf, the *glo *mutant pericardial cavity had become greatly enlarged compared to the control embryos (Figs. 3A and 3D, 4A and 4B).

**Figure 5:**
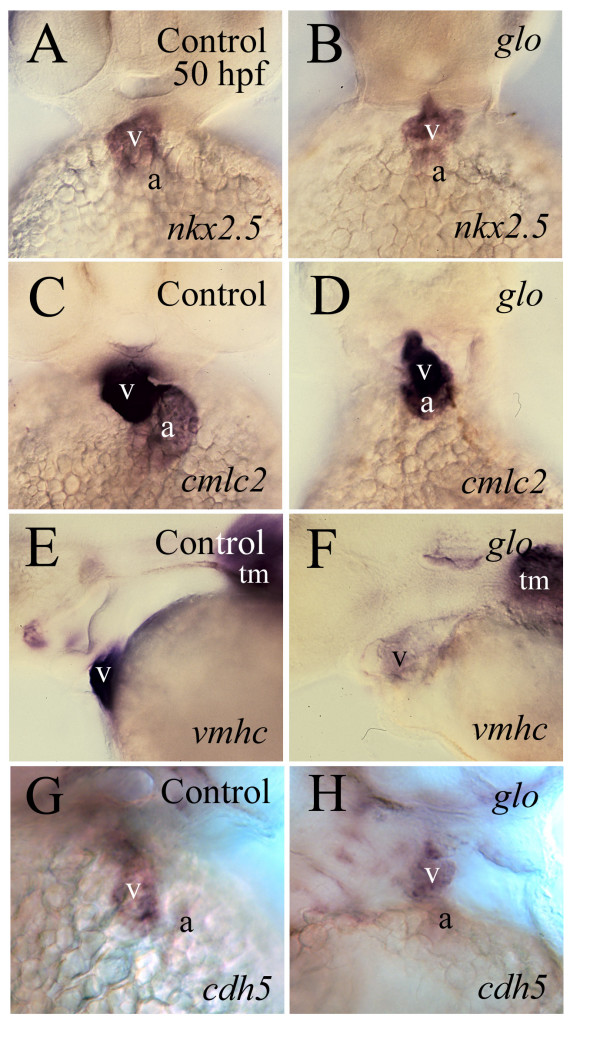
**Expression of cardiac specific genes in control (left column) and *glo *mutant hearts (right column)**. Panels A-D, G and H are ventral views of whole mount hearts with dorsal up. Panels E and F are lateral views of whole mount hearts with anterior to the left and dorsal up. Abbreviation: tm, trunk muscles. Other abbreviations are the same as in Figure 1.

In control 48–50 hpf embryos, the linear cardiac tube, oriented rostrocaudally in younger stages, has developed into the atrium and ventricle, separated by the atrioventricular constriction, with the former located ventrally and to the left of the latter [3,4]. Both the atrium and ventricle are either tubular or oval shaped with smooth surfaces. Gross cardiac morphological defects varied in the *glo *mutant embryos, with some showing tubular, often smaller, atrium and ventricle (Fig. 4B), while others exhibiting irregular shaped atrium and/or ventricle, often with uneven surfaces (Fig. 4C). Moreover, the looping of the heart evident in the control (Fig. 5C) was mostly missing in the mutant heart (Fig. 5D). Some gross cardiac morphological changes were easily detectable at 36 hpf (Fig. 4E). Similar gross cardiac defects were obvious in about half of the *cadherin2 *morphants examined (data not shown), but often with less severity than the *glo *mutants, and no obvious gross cardiac defects were seen in embryos injected with the standard control morpholino oligonucleotide (data not shown) or in the *pac *mutants as reported by [28].

### Differentiation of the *glo *mutant heart is affected

Cardiac differentiation of the *glo *mutants and *cadherin2 *morphants was analyzed using cardiac specific markers *nkx2.5*, *cmlc2*, *vmhc *[34], *cdh5 *[20] or Zn-5 immunostaining. Nkx2.5 is a transcription factor crucial for vertebrate cardiac development [17], and it is expressed mainly by the ventricle at 48–52 hpf (Fig. 5A). Its mRNA expression in the *glo *mutant heart appeared to be similar to the control heart (Fig. 5A and 5B). *cmlc2 *labels the cardiac myosin light chain 2 present in both the embryonic zebrafish atrium and ventricle ([34]; Fig. 5C). Similar to *cmlc2 *expression in the control heart, *cmlc2 *was expressed by both the atrium and ventricle of the *glo *mutant heart (Fig. 5C and 5D). Zebrafish *vmhc *stands for the ventricular myosin heavy chain gene, and it labels the ventricular myocardium and skeletal muscles of the body ([34]; Fig. 5E). *vmhc *expression was moderately or greatly reduced in the *glo *mutant heart, while its expression in their trunk muscle appeared to be less affected (Fig. 5F). *cdh5 *is expressed by zebrafish endothelial layer of the heart throughout embryonic development, with stronger expression in the ventricle than the atrium in two-day old embryos ([20]; Fig. 5G). Expression of *cdh5 *in the *glo *mutant heart was similar to the control (Fig. 5H).

**Figure 6:**
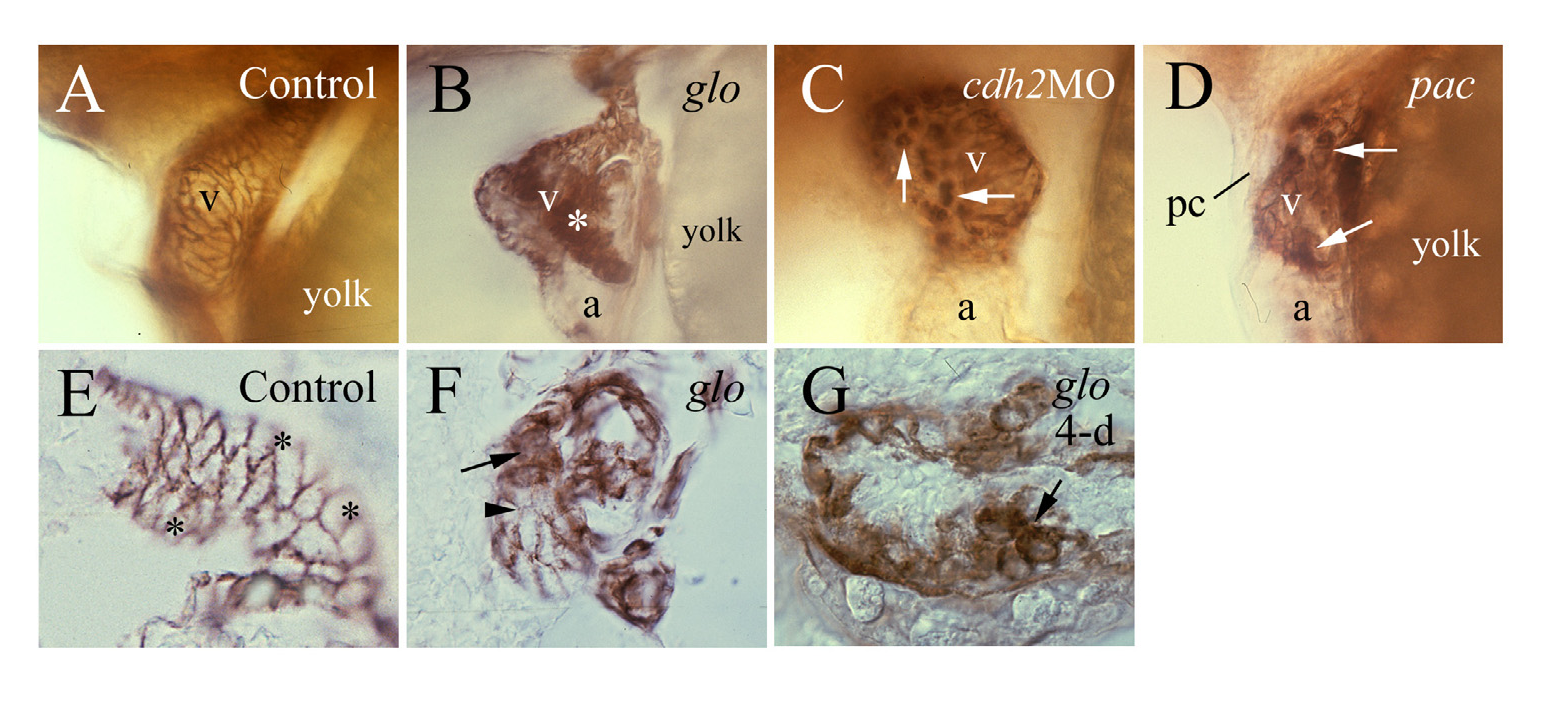
**Zn-5 immunostaining reveals myocardiocyte defectsin a *cadherin2 *morphant, a *pac *mutant embryo and *glo *mutant embryos**. Zn-5 immunostaining reveals myocardiocyte defects in a *cadherin2 *morphant (panel C), a *pac *mutant embryo (panel D) and *glo *mutant embryos (panels B, F and G). All embryos, except panel G (4-day old) are 48–50 hpf. Panels A-D are lateral views of whole mount hearts with anterior to the left and dorsal up. Panels E-G are sections of zebrafish hearts processed for whole mount Zn-5 immunostaining. The asterisk in panel B indicates a large cluster of myocardiocytes with Zn-5 immunoreactivity detected in both their cell membranes and cytoplasm. Arrows in panels C and D point to round shaped myocardiocytes with staining in the cell membrane and cytoplasm. Panel E is a parasagittal section (anterior to the left lower corner and dorsal to the left upper corner) of a control heart. Asterisks in this panel indicate regions that are out of focus. Panel F is a parasagittal section of a *glo *mutant heart (anterior to the left and dorsal up). Panel G is a parasagittal section (anterior to the left and dorsal up) of 4-day old *glo *mutant heart. Arrows in panels F and G point to round shaped myocardiocytes with labeling in their cell membranes and cytoplasm, while the arrowhead in panel F indicates a myocardial cell with weak labeling on its cell membrane.

In the control ventricle, Zn-5 immunostaining was concentrated on the cell membrane of the myocardiocytes (Figs. 1H and 1J, 6E). The ventricular myocardiocytes in control embryos at 48–52 hpf were elongated, fusiform shaped, and form tight associations with one another (Fig. 6A and 6E). Zn-5 labeling of the myocardiocytes in embryos injected with the standard control morpholino oligonucleotide was indistinguishable from that in wild type control embryos (data not shown). Similar to the control heart, Zn-5 immunoreactivity in the *glo *and *pac *mutants and *cadherin2 *morphant hearts was confined mainly to the ventricle (Figs. 4B, 6B–D), but the staining within the mutant and morphant heart ventricles was greatly altered. Zn-5 labeling was detected both on the cell membrane and in the cytoplasm of many *glo *and *pac *mutants, and in *cadherin2 *morphant myocardiocytes (Fig. 6B–D). These cells were often roundly shaped, scattered or formed clusters. These changes were also detected in younger *glo *mutant embryos (Fig. 4E).

### Formation of the vascular system in *glo *mutants is affected

**Figure 7:**
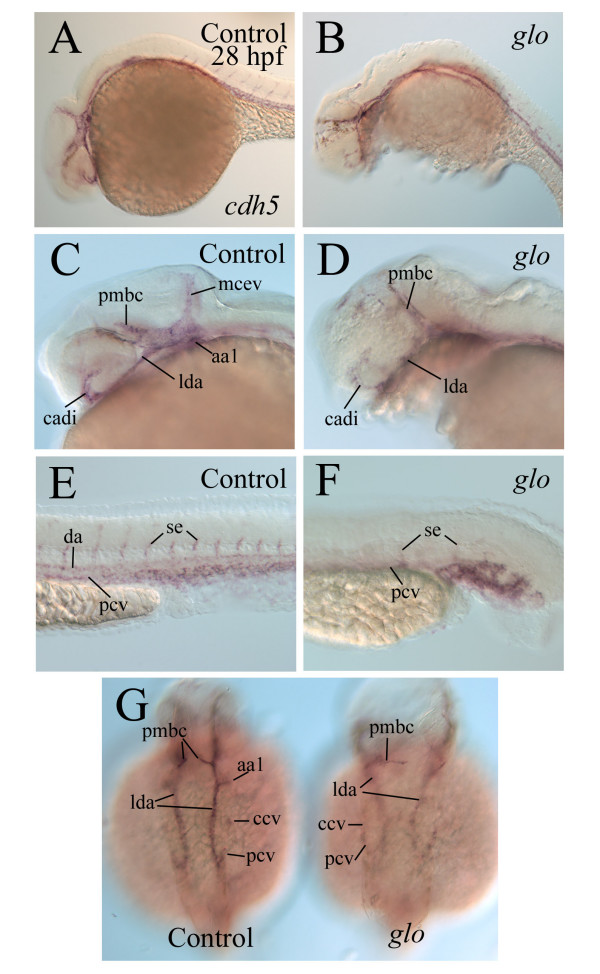
**Development of the vasculature system in 28 hpfcontrol and *glo *mutant embryos as revealed by *cdh5 *staining**. Development of the vasculature system in 28 hpf control (panels on the left column and the embryo on the left in panel G) and *glo *mutant embryos (the remaining panels) as revealed by *cdh5 *staining. Panels A-F show lateral views of whole mount embryos (anterior to the left and dorsal up). Panels C and D are higher magnifications of the head region, while panels E and F are higher magnifications of the trunk and tail regions. Panel G shows dorsal views of the embryo with anterior to the top. Abbreviations: aa1, mandibular arch; cadi, caudal division of the internal carotid artery; ccv, common cardinal vein; da, dorsal aorta; lda, lateral dorsal aorta; mcev, middle cerebral vein; pcv, posterior cardinal vein; pmbc, primordial midbrain channel; se, intersegmental vessel.

**Figure 8:**
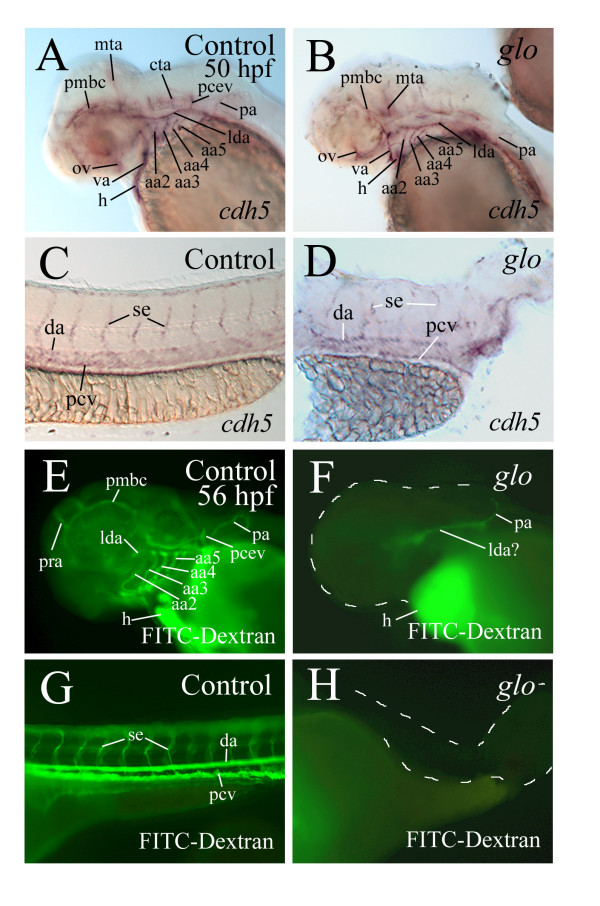
***cdh5 *staining and FITC-dextran labeling of the developing vasculature system in 50 hpf whole mount embryos**. (see Abbreviations) Panels A-D and E-H show *cdh5 *staining and FITC-dextran labeling, respectively, of the developing vasculature system in 50 hpf whole mount embryos (anterior to the left and dorsal up for all panels). Panels in the left column are from control embryos, while panels in the right column are from *glo *mutant embryos. Panels A, B, E and F are lateral views of the head region, while the remaining panels (higher magnifications) show lateral views of the trunk and/or tail regions. The head, trunk and tail of the mutant embryos are outlined by the dashed lines.

The enlarged pericardial cavity in *cadherin2 *morphants and *glo *mutant embryos may be caused by a poorly developed vascular system in these animals, resulting in an accumulation of fluid in the pericardial cavity. We examined vasculature formation in the *glo *mutant embryos using the endothelial marker *cdh5 *(Fig. 7). Although some of the major blood vessels were present at 28 hpf in the mutants, their staining was greatly reduced compared to control embryos (Fig. 7G). Intersegmental vessels in the body and tail are well-developed in control embryos at this stage ([20]; Fig. 7E), but poorly formed in the *glo *mutants (Fig. 7F). By 50 hpf, an elaborate vascular system can be detected using the *cdh5 *staining in zebrafish ([20]; Fig. 8). The majority of the blood vessels seen in control embryos, including the lateral dorsal aorta, dorsal aorta, posterior cardinal vein, hyoid and branchial arch vessels, could also be found in the *glo *mutant embryos (Fig. 8B). The intersegmental vessels in the mutant embryos were poorly stained and disorganized compared to those in control embryos (Fig. 8C and 8D). Injection of FITC-Dextran into anesthetized control embryos resulted in a vasculature labeling pattern similar to *cdh5 *staining in the control embryos (Fig. 8A, 8C, 8E and 8G), but there was very little FITC-Dextran labeling in *glo *mutant embryos (Fig. 8F and 8H).

## Discussion

In this study, we show that zebrafish cadherin2 is expressed by the myocardium of both the atrium and ventricle during critical periods of zebrafish cardiac development, and that loss of cadherin2 function disrupts differentiation of myocardiocytes, normal functioning of the zebrafish heart and formation of the intersegmental vasculature. There are only a few systems (e.g. visual and cardiac systems) and organisms in which one can readily perform *in vivo *studies of gene function in cell and tissue differentiation together with measurement of organ physiological performance affected by the loss of the gene function. This study further demonstrates the usefulness of zebrafish as a model organism to study gene function in vertebrate cardiac differentiation and function. The reduced cardiac performance in the *glo *mutant embryos likely results from altered atrial and ventricular morphology, which in turn is likely caused by the changes in the myocardiocyte differentiation.

### Pericardial and cardiac gross morphology is greatly altered in *cadherin2* morphants and *glo *mutant embryos

Our finding that the pericardial cavity was greatly enlarged in the vast majority of the *cadherin2 *morphants and in all *glo *mutants suggests that cadherin2 function could be required for developing a normal pericardial cavity. However, it cannot be ruled out that altered kidney function could be causing pericardial enlargement via fluid accumulation. Thus, the actual cause(s) for the formation of such an enlarged pericardial cavity, likely due to an accumulation of fluid in the cavity, in these embryos is unclear. It may have more to do with the much-weakened cardiac function in the mutant embryos than the development of the vascular system. This idea is supported by our finding that most of the major blood vessels were present in the *glo *mutant embryos, but there was little labeling of the blood vessels using the FITC-Dextran injection (Fig. 8).

Enlarged pericardial cavity was reported in *cadherin2 *mutant mice [18], but not seen in the *pac *mutants [28]. This difference likely results from different degrees of knockdown/knockout of cadherin2 function in these mutants. It is possible that a small amount of functional cadherin2 protein is produced in the *pac *mutants due to alternative splicing [29], therefore defects in the *pac *mutants are not as severe as in the *glo *mutants.

The gross morphological defects observed in the *glo *mutant hearts are likely caused by changes in the morphology of individual myocardiocytes. Although defects in the myocardiocytes were observed in both *glo *and *pac *mutant embryos, the gross cardiac morphological defects were obvious only in the former. This again, is likely due to differences in the degree of cadherin2 function disruption, with a complete loss of cadherin2 function in the *glo *mutant embryos, while perhaps some cadherin2 function remaining in the *pac *mutant embryos (see above). The enlarged pericardial cavity is unlikely one of the major causes of the atrial and ventricular disorganization, because the gross morphological defects can be detected at 36 hpf, when there is no obvious change in the size of the pericardial cavity.

### Cadherin2 plays an important role in myocardiocyte differentiation, cardiac morphogenesis and performance

During cardiac development, myocardiocytes express cardiac specific markers such as *nkx2.5*, *cmlc2*, *vmhc *and *cdh5*[3,20]. Despite greatly altered gross cardiac morphology and myocardiocytes morphology in *glo *mutant embryos, expression of *nkx2.5*, *cmlc2 *and *cdh5 *is largely unchanged, suggesting that cadherin2 is not required for normal expression of these genes. However, expression of *vmhc*, a ventricle specific gene, in the *glo *mutant heart is reduced compared to control embryos. Although vmhc role in cardiac formation and function has not being studied in vertebrates, loss of function in an atrium specific myosin heavy chain has been linked to disruption in atrial function and altered ventricular morphogenesis in zebrafish [35], suggesting that vmhc may play a similar role in ventricular myocardiocyte differentiation and function. Therefore, it is reasonable to speculate that cadherin2 function in vertebrate cardiac development and the function may be mediated, at least partially, by its effect on *vmhc *expression.

Myocardiocytes undergo extensive morphological changes during cardiac morphogenesis [36]. For example, cuboidal shaped chicken myocardiocytes become flattened and tightly associated when the heart begins to contract, and the myocardiocytes become fusiform shaped and arranged in the circumferential direction during looping [36], similar to the zebrafish myocardiocytes (Fig. 6A and 6E). Many myocardiocytes in the *glo *mutant become round shaped and lack tight association, which may have contributed to poor electrical conduction between cells, thus reducing the contraction rate shown in *glo *mutant embryos. This also may have contributed to the gross cardiac morphological defects including the disrupted heart looping observed in both the cadherin2 mutant mice [18] and *glo *mutant embryos.

The loss of cadherin2 function does not affect all aspects of myocardiocyte differentiation because myocardiocytes in the cadherin2 mutant mice and *glo *mutant embryos still contract and/or express cardiac specific genes (see above). Other cell adhesion molecules such as N-CAM and other cadherin molecules may still function in these animals. Although most of the blood vessels were present as indicated by *cdh5* labeling, it is unclear whether or not these *cdh5*-positive vessels were normal. As was shown in the heart, *cdh5* staining in the *glo *mutants was similar to controls. It is not surprising that the formation of the major trunk vessels is not greatly affected in the *glo *mutant embryos, since the zebrafish vasculature system express *cdh5*, instead of cadherin2. It is possible that there are defects on those blood vessels, but we have no other markers (e.g. *Zn-5*, *vmhc*) to assess their integrity. Additionally, the intersegmental vessels and all other vessels formed via angiogenic remodeling are poorly formed or not present in *glo *mutant embryos. This is likely a proximal effect of the lack of pressure and flow generated by the *glo *mutant embryo heart, and/or due to cadherin2 function on the trunk and tail muscle development [37].

## Conclusion

Our results suggest that cadherin2 plays an essential role in zebrafish cardiovascular development. Although the exact mechanisms remain unknown as to the formation of the enlarged pericardium and reduced peripheral blood flow, it is clear that myocardiocyte  differentiation and physiological performance is impaired.

## Methods

### Zebrafish

Zebrafish (*Danio rerio*) were maintained at 28.5°C as described in the Zebrafish Book [38]. The *glo *heterozygous mutant carriers and their wildtype siblings from a single breeding, obtained from the Zebrafish International Resource Center at the University of Oregon (Eugene, OR) as embryos, were raised to reproductive maturity in the animal care facility at the University of Akron. Pair-wise breeding was performed to identify *glo *heterozygous mutant carriers, and the *glo *mutant embryos were identified by gross morphological phenotype. Their wildtype and heterozygous siblings were used as controls. Embryos for cadherin2 morpholino oligonucleotides (MO) experiments were obtained from breeding of wildtype adult zebrafish. Zebrafish embryos homozygous for the *pac *mutation (*pac*^*tm101B*^) were obtained from Max-Planck Institute for Developmental Biology (Tübingen, Germany). Embryos for whole mount *in situ *hybridization were raised in PTU (1-phenyl-2-thiourea, 0.003%) in order to reduce optical interference of pigments. All animal-related procedures were approved by the Care and Use of Animals in Research Committee at the University of Akron.

### Measurement of cardiac performance in control and *glo* mutant zebrafish embryos

*glo *mutant (*N *= 10) and control zebrafish embryos (heterozygous and wildtype siblings of the *glo *embryos, *N *= 8) were used for measuring cardiac function. Mutant embryos were identified and separated from wild type embryos using a Leica dissecting microscope, based on morphological differences such as curved spines and tail blisters [29]. The embryos were dechorionated at approximately 24 hpf and were kept in a small plastic compartmentalized container (2.5 ml for each compartment) with tank water at 28.5°C for the duration of the experiment. Immediately before each measurement, the embryos were immobilized using MS-222 (0.02%). At this early stage of development and at this low concentration of MS-222, there are no measurable effects of this anesthesia on the cardiovascular system (unpublished data). The embryos were digitally recorded at 30 hpf, 36 hpf, 48 hpf, 58 hpf, and 72 hpf using a temperature controlled inverted microscope (Leica, DMIRB) equipped with a digital video camera (Redlake MASD, San Diego, CA). For each embryo at each selected period of development, a 10 sec digital video was captured at 0.008 second intervals (125 frames per second). At the completion of each video, the individual fish were returned to their respective compartments and were tracked throughout the experiment.

Videos were analyzed for heart rate, end diastolic volume, end systolic volume, stroke volume, and cardiac output using ImagePro Plus^® ^imaging software (Media Cybernetics, Silver Spring, MD). Heart rate was calculated by counting the number of sequential contractions, beginning and ending at end diastole, occurring in the video file and dividing by the exact time interval. End diastolic volume was determined by measuring the perimeter of the ventricle at diastole (obtained by tracing the ventricle in a single frame of the cardiac cycle video stopped where the ventricle was at its largest point) along with the length and width of the ventricle at diastole. End systolic volume was determined by measuring the perimeter of the ventricle at systole (where the ventricle was at its narrowest width following diastole) in addition to the length and width of the ventricle at systole. The resulting ventricular volumes were calculated using the formula (8/3π) *a*/L, where *a *is the area of the traced ventricle and L is the length of the ventricle at either diastole or systole [39]. The stroke volume was calculated by subtracting the end systolic volume from the end diastolic volume and the cardiac output was calculated by multiplying the stroke volume by the heart rate. Data were analyzed for differences between the *glo *and control embryos and over development time by using a two-way repeated measure ANOVA. Post hoc comparisons were performed using Tukey's multiple comparisons procedure. All physiological data presented are means ± S.E.M.

### MO and FITC-dextran Injections

A cadherin2 translation blocking morpholino oligonucleotide (5'-TCTGTATAAAGAAACCGATAGAGTT-3', [28], or a standard control (5'-CCTCTTACCTCAGTTACAATTTATA-3'), gifts from Dr. James Marrs (Indiana University) who purchased it from Gene Tools, Corvallis OR, was microinjected into either blastomeres and/or the yolk immediately below the blastomeres of 1–4 cell stage wild type embryos. Injected embryos were allowed to develop at 28.5° C until desired stages. Fluorescein isothiocyanate (FITC)-dextran (Sigma) was injected into the common cardinal vein of anesthetized embryos (56 hpf) according to [40].

### Tissue processing

Zebrafish embryos were euthanized in 0.02% methane tricaine sulfonate (MS-222, Sigma, St Louis, MO) and fixed in 4% paraformaldehyde in 0.1 M phosphate buffered saline (PBS) overnight at 4°C. To prepare tissue for whole mount *in situ *hybridization or immunohistochemistry, the tissue was rinsed in PBS, followed by 70% methanol and 100% methanol. The tissue was stored in 100% methanol at -20°C. Preparation of tissues for immunohistochemical staining on sections was described previously [41]. Briefly, the fixed tissue was processed through a graded series of increasing sucrose concentrations, placed in 20% sucrose in PBS overnight, then embedded and frozen in a mixture of OCT embedding compound and 20% sucrose (1:1, v/v). A cryostat was used to obtain 12–14 μm sections. Some *glo *and control embryos processed for whole mount *in situ* hybridization or immunostaining were embedded and sectioned as described above. Tissue sections were collected on pretreated glass slides (Fisher Scientific, Pittsburgh, PA), dried at room temperature and stored at -80°C.

### *In situ* hybridization

A cDNA containing the presequence, the extracellular and transmembrane domains of zebrafish *cadherin2*, obtained by RT-PCR from 24 hpf embryonic zebrafish total RNA, was used as a template to generate anti-sense and sense *cadherin2 *cRNA probes [42]. cDNAs used to generate zebrafish *nkx2.5*, *cardiac myosin light chain 2 *(*cmlc2*), and ventricular myosin heavy chain (*vmhc*) cRNA probes were kindly provided by Deborah Yelon at the New York University [34]. Zebrafish *cdh5 *cDNA used to generate *cdh5 *cRNA probes was kindly provided by Jon Larson at the Discovery Genomics [20]. Synthesis of digoxigenin-labeled cRNA probes, procedures for whole mount *in situ *hybridization were described previously [41]. Anti-digoxigenin Fab fragment antibodies conjugated to alkaline phosphatase were used for immunocytochemical detection of the cRNA probes, and this was followed by an NBT/BCIP color reaction step (Roche Molecular Biochemicals, Indianapolis, IN).

### Immunohistochemistry

Procedures for whole mount immunohistochemistry and immunostaining on tissue sections were described in detail previously [43,44]. Primary antibodies used were affinity purified zebrafish cadherin2 antibody (1:80, [43]) and Zn-5 (1:1000, Zebrafish International Resource Center, University of Oregon, Eugene, OR). Biotinylated secondary antibodies (Vector Laboratories, Burlingame, CA) were used at 1:200. Visualization of the reaction was achieved by using a DAB kit (Vector Laboratories). For immunofluorescent double labeling experiments, the secondary antibodies were Cy3-conjugated anti-rabbit IgG and FITC-conjugated anti-mouse IgG (Jackson ImmunoResearch Laboratories, West Grove, PA) for the detection of cadherin-2 and Zn-5, respectively.

## Abbreviations

aa2, hyoid arch; aa3, first branchial arch; aa4, second branchial arch; aa5, third branchial arch; cta, central artery; h, heart; mta, metencephalic artery; ov, optic vein; pa, pectoral artery; pcev, posterior cerebral vein; pra, prosencephalic artery; va, ventral aorta. Other abbreviations are the same as in figure

## Authors' contributions

BB participated in the design of the study, executed some of the experiments, collected and organized the physiological data, and was involved heavily in the editing process and the final draft production. JF participated in the design of the study and was heavily involved in executing most of the experiments and analyzing the data. JF was also heavily involved in the rough draft of the manuscript. BL was involved in the design of the study and was heavily involved in the experimental aspects of the project. QL was the overseer of the entire project and was involved at all levels. QL had heavy input into both the rough and final versions of the manuscript.
